# Adenomatoid tumors of ovary mimicking malignancy: report of 2 cases and literature review

**DOI:** 10.1186/s12905-022-02138-6

**Published:** 2022-12-26

**Authors:** Lili Sun, Zehua Zhao, Ning Qu, Yanmei Zhu

**Affiliations:** 1 Department of Pathology, Cancer Hospital of Dalian University of Technology, Cancer Hospital of China Medical University, Liaoning Cancer Hospital and Institute, 44 Xiaoheyan Road, Dadong District, Shenyang, 110042 Liaoning China; 2 Department of Radiology, Cancer Hospital of Dalian University of Technology, Cancer Hospital of China Medical University, Liaoning Cancer Hospital and Institute, 44 Xiaoheyan Road, Dadong District, Shenyang, 110042 Liaoning China

**Keywords:** Adenoatoid tumor, Ovary, Laparoscopic surgery, L1CAM, BAP1, CDKN2A/p16

## Abstract

**Background:**

Adenomatoid tumors (ATs) are benign tumors originating from the mesothelium. ATs of the ovary are rare, and can easily be confused with malignancy due to the histomorphological diversity. Thus, it is difficult in histopathological and differential diagnosis, especially during intraoperative frozen pathological diagnosis, which directly affects the resection scope of surgery.

**Case presentation:**

In this study, we reported two patients (58 and 41 year old) with ovarian ATs. AT of patient 1 occurred in both ovaries at different time points and she had been diagnosed with Hashimoto's thyroiditis. AT of patient 2 occurred in right ovary. Intraoperative frozen pathological diagnosis was performed in both cases and laparoscopic salpingo-oophorectomy was undergone on the lesion side according to benign freezing diagnostic result. Ovarian ATs, the final diagnoses of the 2 cases were concluded after histological, extensive immunohistochemical (IHC), histochemical, and fluorescence in situ hybridization analyses.

**Conclusions:**

Our results show that ovarian ATs may not be related to BAP1 or CDKN2A/p16 mutations. In addition, the case 1 suggests that ATs may be associated with immune dysregulation. When encountering such similar lessions, we recommend that a series of immunohistochemical, histochemical and molecular biological techniques should be used for diagnosis and differential diagnosis to avoid misdiagnosis. Improving understanding of the rare ovarian ATs which mimic malignancy is necessary to prevent overresection.

## Background

Adenomatoid tumors (ATs) belong to a family of benign tumors that originate from the mesothelium [1, 2]. In 1916, Sakaguchi first reported ATs as “adenomyomatoma” [3]. Initially, their histogenesis remained unclear until several studies identified their origin to be the mesothelium [1, 2, 4], which is now widely accepted.

ATs mainly occur in the male and female genital tracts, and rarely in extragenital regions, such as serosal membrane sites (pleura, peritoneum, and pericardium), adrenal glands, and visceral organs [1]. In the female genital tract, ATs rarely appear in the ovary, but often involve the uterus and fallopian tube [5].

Histological growth patterns of ATs mainly include adenoid, angiomatoid, cystic, solid, tubular, and various combinations of these main patterns [1, 2, 6]. Due to the diverse histomorphological features of ATs, the histopathological and differential diagnoses of ATs are often difficult to make. In the case of ATs in the ovaries, diagnosis is most especially difficult due to the few case reports in literature. Intraoperative frozen pathological diagnosis of ovarian ATs is even more challenging. Accurate qualitative diagnosis will directly affect the scope of laparoscopic resection.

In this study, we described two cases of ATs in the ovaries and reviewed the literature. It will further improve our understanding of the histopathological, immunohistochemical (IHC), and molecular genetic features of this rare tumor.

## Methods

### Immunohistochemical stain

CK, ER, PR, AR, P16, and Ki67 were performed using an automated system (Benchmark XT, Roche Ventana, Tucson, AZ, US). Other antibodies were carried out according to GTVision™ Kit instructions (GK600711, Gene Tech, Shanghai, China). Antigen extraction was performed for each antibody according to the manufacturer's instructions. Nonspecific stain blocker was used to block endogenous peroxidase activity. Sections were incubated with each antibody and HRP enzyme-conjugated goat anti-mouse/rabbit IgG polymer. 3.3′-diaminobenzidine (DAB) was used to develop the color, then hematoxylin was used to counterstain.

### Histochemical stain

Alcian blue and periodate Schiff's reaction (AB-PAS) staining fluid (BA4121, BaSO Companh, Zhuhai, China) was used for histochemical stain. Sections were routinely dewaxed to water and washed with distilled water, dyed with alcian blue staining fluid (PH2.5) for 10–20 min, washed with water and removed excess water, oxidized by periodic acid solution for 10 min, rinsed with distilled water and removed excess water,dyed with schiff reagent for 10–15 min, rinsed with water and removed excess water, dyed the nucleus with hematoxylin solution for 2–3 min, rinsed with water and dried excess water,finally sealed with neutral gum after conventional dehydration transparency.

### FISH detection

CDKN2A/p16 (9p21) two-color fluorescence deletion probe kit (F.01265-01, Anbiping Company, Guangzhou, China) was applied for FISH. Tissue sections were pretreated with pure water at 88–92 °C for 30 min and digested with pepsin at 37 °C for 15–20 min after baking and dewaxing, then further rinsed, fixed, and dehydrated. 10 μL of the probe working solution was placed onto the tissue specimen and the edges sealed with rubber cement after the tissue sections naturally drie. Tissue sections were put in a hybridization apparatus, denatured at 78 °C for 5 min, and hybridized at 42 °C for 12–18 h. Tissue sections were washed, dried and added 10 μL 4′,6-diamidino-2-phenylindole after hybridization, then put at room temperature for 15 min. The BioView automatic scanning image analysis system (Duettm) was used to store and analyze the fluorescence FISH images. Finally, the FISH slides were stored at − 20 °C in the dark for future experiments. For each slide, 100 nuclei of tumor cells were analyzed with two observers. 0 red and 2 green represented homozygous deletion, which were considered positive. When the cut-off was greater than or equal to 10%, the specimen was diagnosed as CDKN2A/p16 (9p21) gene deletion.

### Case presentation

#### Case 1

**Fig. 1:**
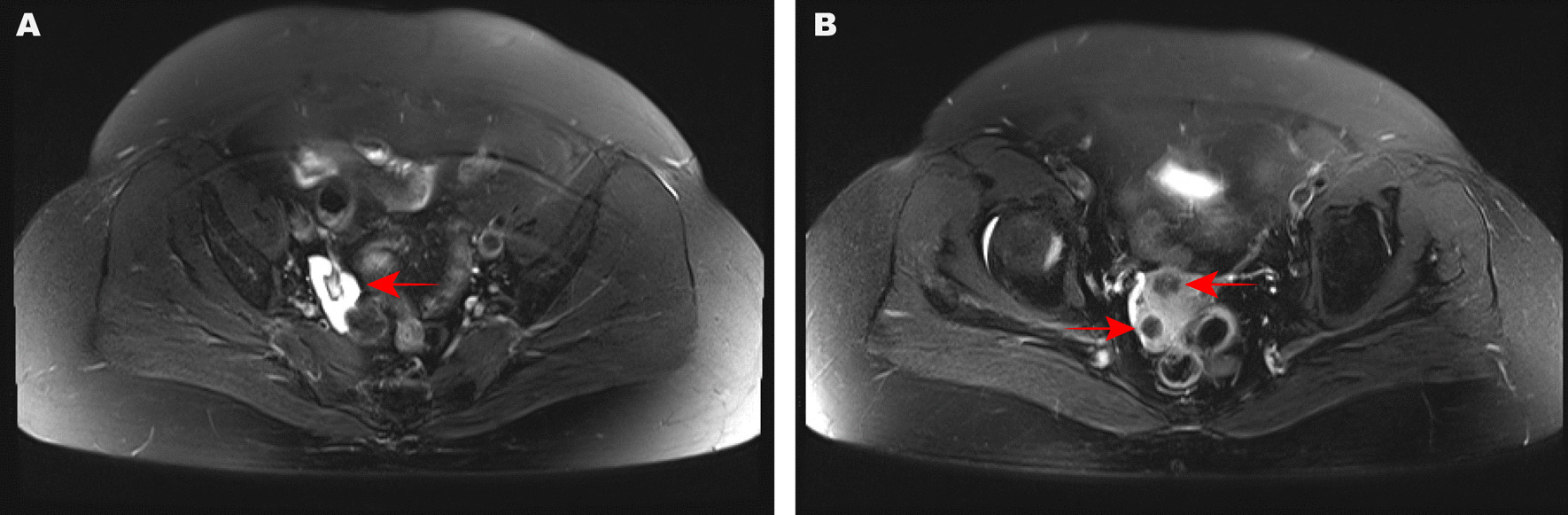
MRI performance. **A** A mass in the right adnexal area. **B** Multiple nodules in the uterus

A 58-year-old woman presented with a one-month history of intense abdominal pain. Abdominal ultrasound, computed tomography (CT), and magnetic resonance imaging (MRI) all suggested the presence of a 3-cm long mass in the right adnexal area (Fig. 1a) and multiple uterine nodules (Fig. 1b). The mass in the right adnexal area was cystic and solid, and had enhancement due to tumor vascularity from MRI, which suggested malignant potential. The patient had elevated levels of c-reactive protein (CRP, 48.75 mg/L) and thyroid-stimulating hormone (TSH, 6.22 uIU/mL). The plasma levels of tumor markers, including CA125, HE4, CA199, CA724, CEA, CA153, SCC, AFP, AFU, and NSE, were normal. Her medical and surgical history revealed that she had been diagnosed with Hashimoto's thyroiditis > 30 years ago, undergone a minor surgery to remove a laryngeal polyp 15 year ago, and undergone laparoscopic left salpingo-oophorectomy 8 years ago. She had a 30-year history of smoking 10 cigarettes daily, but no history of alcohol consumption. The patient had had 11 pregnancies in her lifetime, 10 of which resulted in miscarriages (G11P1).

Macroscopically, the dimensions of the right ovariectomized specimen were 3 cm × 3 cm × 2 cm. The specimen had a partial defect, which resulted from the intraoperative frozen examination of the specimen. The central area of the specimen, which comprised normal ovarian tissue measuring about 2 cm × 1.5 cm × 1 cm, was tough with a porcelain white color. The tumor was present on the circumferential surface. The mass had a greyish-yellow color. A cut surface of the tumor revealed a mucus-containing honeycomb-like structure with a soft texture.

**Fig. 2:**
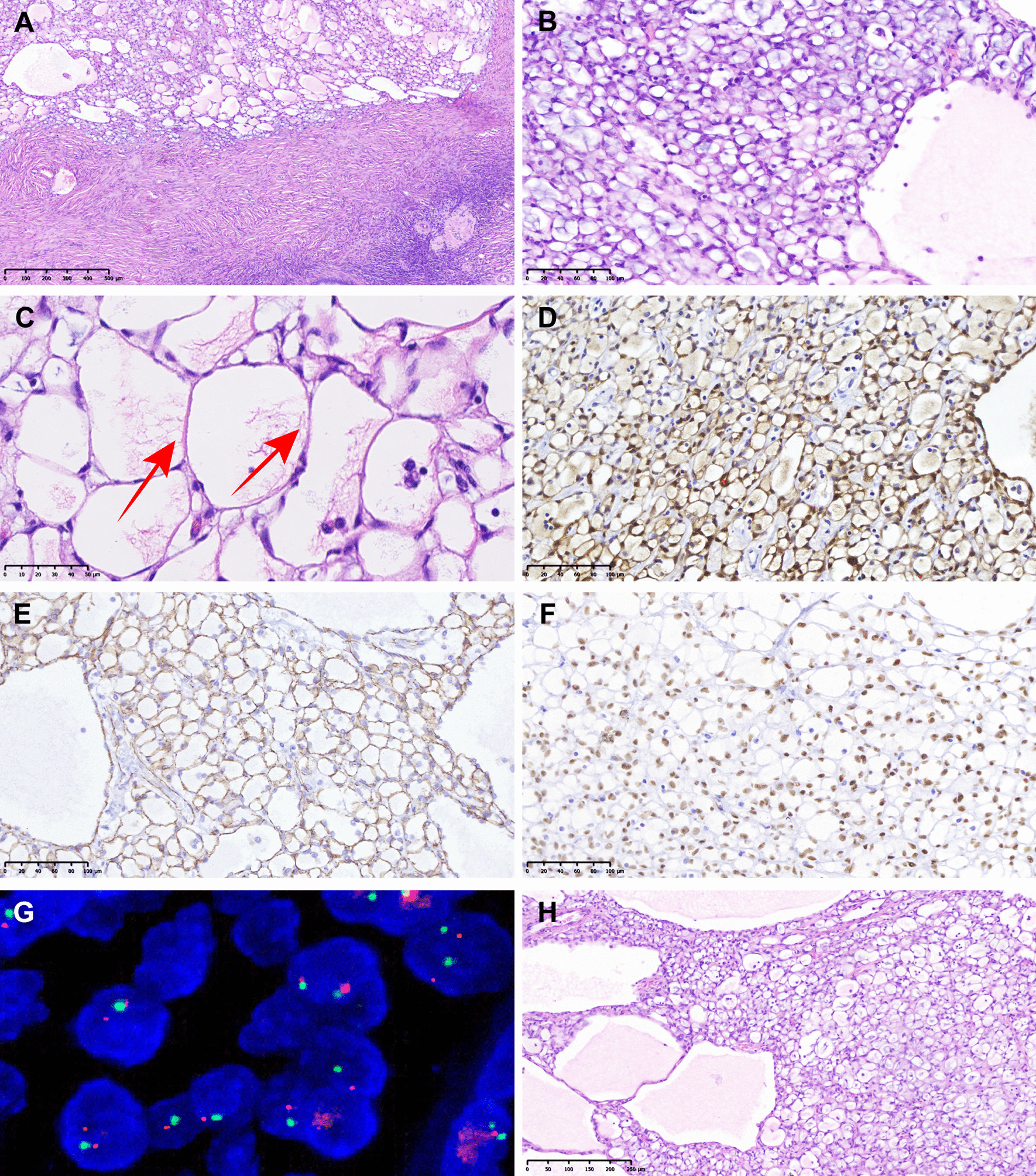
Morphological characteristics in both bilateral ovaries (**A–C**: Right; **H**: Left), Immunohistochemical (× 200) and FISH analyses of Case 1. **A** Well-defined from ovarian parenchyma (H&E, × 50). **B** Vacuolated cytoplasm and Signet-ring cells (H&E, × 200). **C** Thread-like bridging strands (H&E, × 400). **D** Diffuse strong positive for Calretinin. **E** Diffuse strong positive for L1CAM. **F** Diffuse strong positive for BAP1. **G** CDKN2A/p16 gene deletion negative. **H** Adenoid, cystic and solid growth patterns (H&E, × 100)

Microscopically, the histological morphology of intraoperative frozen pathological and postoperative paraffin sections was basically the same. At low magnification, the tumor tissue had an interspersed distribution of macrocystic, microcystic, and solid growth patterns, which were well-defined from normal ovarian parenchyma (Fig. 2a). At high magnification, the tumor tissue comprised multiple mutually anastomosing adenoid spaces, which were lined with flat, cubic, or short columnar epithelioid cells with mild atypia. The cell cytoplasm was vacuolated, and occasionally contained basophilic substances. Signet-ring cells were seen locally (Fig. 2b). Thread-like bridging strands were present within the luminal spaces (Fig. 2c). Hyaline connective tissues were present in trace quantities. No eosinophilic cytoplasm and inflammatory lymphocytic infiltration were present in this case.

Because the patient had a history of laparoscopic left salpingo oophorectomy, and morphology lacked of evidence of malignant tumor, such as obvious cell atypia and invasive growth pattern, it was considered as a benign lesion. Subsequently, she underwent laparoscopic hysterectomy and right salpingo-oophorectomy based on intraoperative pathological diagnosis.

The IHC analysis revealed that CK, CK7, Calretinin (Fig. 2d), WT-1, D2-40, L1CAM (Fig. 2e), and BAP1 (Fig. 2f) were fully expressed. CK5/6 and Cyclin D1 were focally expressed; β-catenin was fully expressed in the membrane. CD56 was focally expressed, and P16 was patchy positive. CD34 had a positive expression in vessels, and Ki67 was about 3% positive. However, EMA, CEA, MOC-31, Ber-EP4, Vimentin, SALL4, inhibin-α, SF-1, CD10, CD99, S-100, SMA, MelanA, PAX-2, GATA-3, ER, PR, AR, and CK20 were negative. AB-PAS staining revealed an AB-positive stain. Upon FISH analysis, CDKN2A/p16 gene deletion test was shown to be negative (Fig. 2g).

Based on the histomorphological, IHC, histochemical, and FISH detection findings, a final diagnosis of AT of the right ovary and multiple leiomyoma of the uterus was made. From her medical history, she had already been diagnosed with AT of the left ovary after multiple hospital consultations. The microscopic appearance of the left ovary is shown in Fig. 2h. The histomorphological features of both ovaries were similar. The present postoperative follow-up time was 13 months, and no recurrence or metastasis was found.

#### Case 2

A 41-year-old woman was found to have a mass in her right adnexal area during a physical examination, which had been gradually increasing in size for two years. Ultrasound and CT of the abdomen suggested the presence of a 3-cm long mass in the right adnexal region, which was also cystic and solid, and color dopper flow image (CDFI) from ultrasound showed increased flow signals. Plasma levels of tumor markers were normal. The patient had no prior medical or surgical history, and no history of smoking or alcohol consumption.

Macroscopically, the diameter of the removal right ovarian mass specimen was approximately 2.5 cm. The specimen was partially defective due to intraoperative frozen sampling. The cut surface was alternately cystic and solid. The solid area was 1.5 cm in diameter, yellowish white in color and soft in texture.

**Fig. 3:**
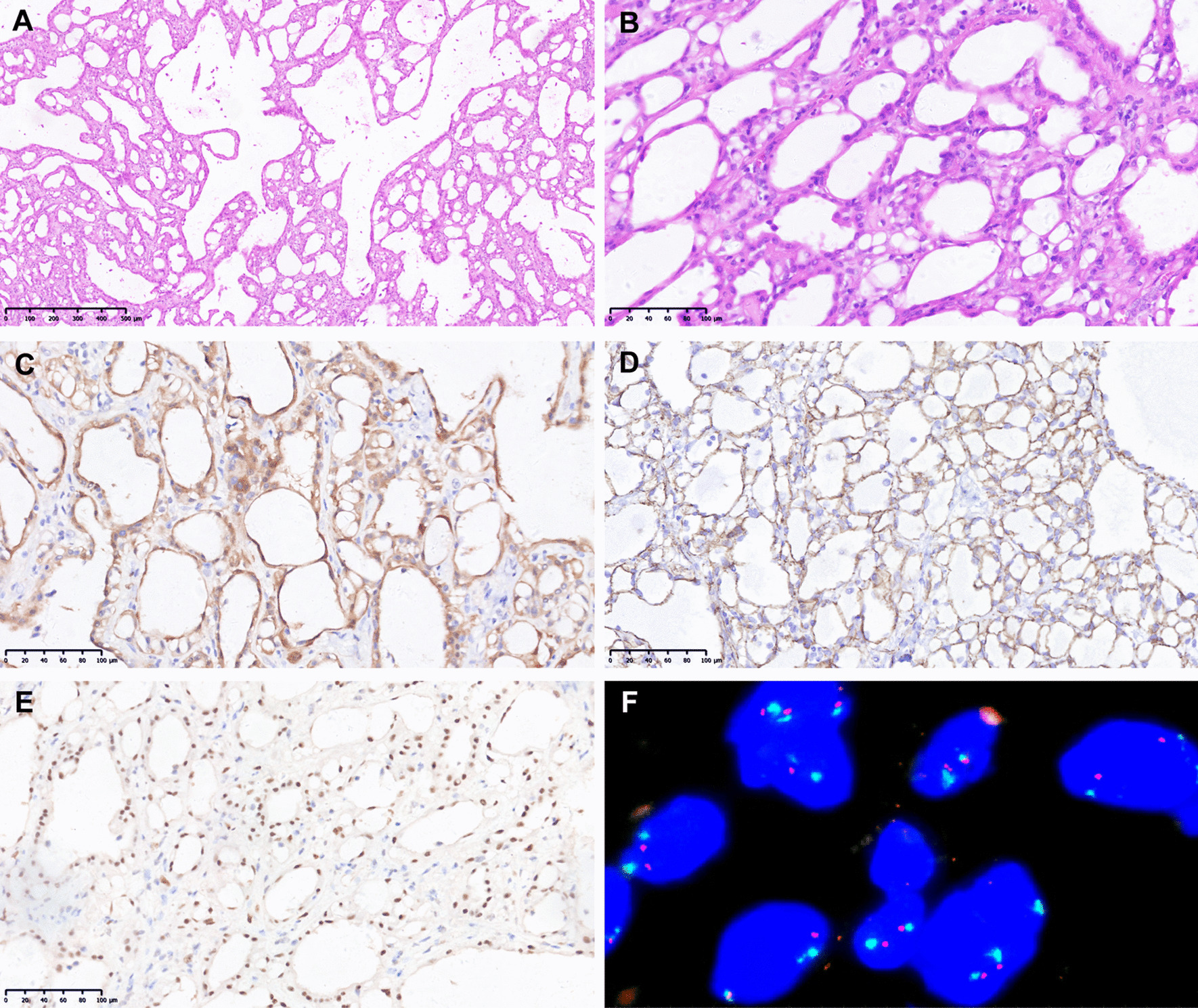
Morphological characteristics, Immunohistochemical (× 200) and FISH analyses of Case 2. **A** Cysts, adenoid and solids growth patterns (H&E, × 50). **B** Vacuolated cytoplasm, signet-ring cells, and thread-like bridging strands (H&E, × 200). **C** Diffuse strong positive for Calretinin. **D** Diffuse strong positive for L1CAM. **E** Diffuse strong positive for BAP1. **F** CDKN2A/p16 gene deletion negative

Microscopically, the tumor tissue showed multiple growth patterns such as large cysts, small cysts, adenoid and solids under low magnification (Fig. 3a). The tumor cells were flat, cuboidal or short columnar epithelioid cells with mild to moderate cellular atypia under high magnification. The cell cytoplasm was eosinophilic and occasionally vacuolated. Signet-ring cells and thread-like bridging strands were seen locally (Fig. 3b). There was a certain amount of hyaline stroma and no inflammatory lymphocytic infiltration. The intraoperative frozen pathological diagnosis was benign lesion, and she subsequently underwent laparoscopic right salpingo-oophorectomy.

The IHC analysis revealed that CK, CK7, Calretinin (Fig. 3c), WT-1, CK5/6, D2-40, L1CAM (Fig. 3d), and BAP1 (Fig. 3e) were fully expressed. Cyclin D1 and CD56 were focally positive. β-catenin was fully expressed in the membrane. P16 was patchy positive. CD34 had a positive expression in vessels, and Ki67 was approximately 5% positive. However, EMA, CEA, MOC-31, Ber-EP4, Vimentin, SALL4, inhibin-α, SF-1, CD10, CD99, S-100, SMA, MelanA, PAX-2, GATA-3, ER, PR, AR, and CK20 were negative. AB-PAS staining revealed an AB-positive stain. CDKN2A/p16 gene deletion was negative by FISH analysis (Fig. 3f). The postoperative follow-up time was 26 months, and no recurrence or metastasis was found.

## Discussion

ATs are benign neoplasms originating from the mesothelium of the female and male genital tracts in most cases [1]. In the female genital tract, the uterus [7] and fallopian tube [8] are the most common sites for ATs. ATs rarely occur in the ovary [5]. Few well-documented reports of rare cases of ovarian ATs were present in English literature [5, 9–12], and they have been summarized in Table 1.

**Table 1:** Clinicopathologic features summaries of the ovary adenomatoid tumors in the literature

Case no.	Author	Age (years)	Laterality	Location	Size (mm)	Definite symptoms	Gross examination	Lymphocytes infiltration	AB-PAS stain	Ultra-structure	IHC
1	Hirakawa et al	61	Left	Hilus	8 × 7	No	Gray-white, cystic and solid	Yes	AB+, PAS−	Mesothelial-like	CK,CA125,Vim+; FVIII, UEA I, CEA, EMA−
2	Ghossain et al	69	Left	NA	40 × 30	Yes	Multilocular cystic	Yes	NA	NA	CK+; CD34−
3	Young et al	23–79	NA	Hilus	1	No	Most solid	1/3 Yes	NA	NA	NA
4					2	No					
5					4	No					
6					7	No					
7					14	Yes					
8				Juxtaovarian	50	NA					
9	Phillips et al	52	Right	Most replaced	50	No	White–yellow, solid	Yes	AB+, PAS−	NA	AE1/3, Calretinin, WT-1+; CK5/6 focal+; Ber-EP4, CEA−
10	Shi et al	44	Right	Adjacent ovarian	8 × 8 × 7	No	Solid	Yes	NA	NA	NA

+, Positive; −, negative; NA, not available

The tumor cells were initially proposed to originate from mesothelial, endothelial, mesonephric, primitive Müllerian pluripotent mesenchymal, or coelomic epithelial cells [13, 14]. However, Masson in 1942 and Evans in 1943 reported the tumor cells had mesothelial differentiation [15, 16]. In 1945, Golden and Ash proposed the descriptive term, “adenomatoid tumor” [17]. Subsequently, several studies confirmed the mesothelium as the origin of ATs using histological, immunophenotypic, and ultrastructural analyse [1, 2, 4].

Recent reports indicate that ATs genetically harbor somatic missense mutations in the TRAF7 gene (encoding an E3 ubiquitin ligase belonging to the family of tumor necrosis factor receptor-associated factors (TRAFs), which activates the NF-κB pathway and increases expression of L1CAM, a marker of NF-kB pathway activation [18, 19]. Additionally, ATs uniformly lack BAP1, CDKN2A, and NF2 mutations; similarly, well-differentiated papillary mesothelial tumors lack these mutations. IHC demonstrates intact nuclear expression of BAP1 and robust membrane expression of L1CAM in ATs [20, 21]. Unlike in ATs, the *BAP1* tumor suppressor gene mutation has been defined as a frequent genetic alteration in mesotheliomas, and an associated loss of nuclear BAP1 immunostaining has been shown to be present in more than 80% of multiple case series [22–28].

The diagnosis of ovarian ATs bilaterally at different time points is noteworthy in case 1. Her medical and drug histories of Hashimoto's thyroiditis and long-term immunosuppressive therapy may underlie the numerous miscarriages (G11P1). Some studies found that ATs occur in immunocompromised individuals, such as patients undergoing immunosuppressive therapy after kidney transplantation and patients with chronic hepatitis C virus infection [29–33]. This suggests a potential link between ATs and immune dysregulation [20]. ATs are hypothesized to be an immunosuppression-induced disease. Other theories propose that an immunosuppressed state promotes ATs development. Further studies are needed to elucidate the exact mechanism.

Uterine and fallopian tube ATs are easier to diagnose than ovarian ATs, because of the prominent smooth muscle components in the uterus and fallopian tubes. The diverse growth patterns, including adenoid, cystic, and solid patterns, as well as the presence of signet ring cells complicate the diagnosis of ovarian ATs, because many ovarian tumors have similar histological characteristics. It is important to emphasize that morphology of ovarian ATs could mimic malignancy. Therefore, misdiagnosis especially during intraoperative frozen pathological diagnosis can lead to overresection. The differential diagnoses of ovarian ATs are summarized in Table 2.

**Table 2:** Morphophenotypic features summaries in differential diagnoses of the ovary adenomatoid tumors

Similar neoplasms	Morphological features	IHC markers	Histochemical stain	Molecular pathology
Adenomatoid tumors	Various growth patterns, signet ring and vacuolated cells with mild to moderate atypia	CK, CR, CK5/6, WT-1, D2-40, L1CAM, BAP1+; Ber-EP4, MOC31, ER, PR −	AB+; PAS−/weak+	Lack BAP1 mutation and CDKN2A/p16 delection
Krukenburg tumors	Signet ring cells and various other architectural patterns with a variably conspicuous stromal component	CK, EMA+	Mucin+	NS
Well-differentiated papillary mesothelial tumors	Common patterns: papillary, tubulopapillary, adenomatoid-like and branching cords, cells with rare mitoses	CK, CR, CK5/6, WT-1, D2-40, L1CAM, BAP1+; EMA+/−; Ber-EP4,MOC31,ER,PR −	NS	Lack BAP1 mutation and CDKN2A/p16 delection
Mesotheliomas	Common patterns: tubular, papillary and solid, cells with mild to moderate nuclear atypia and variable mitotic activity	CK, CK7, CR, CK5/6, WT-1, D2-40+; EMA+/−; L1CAM, BAP1, Ber-EP4, MOC31, ER, PR−	NS	Harbor BAP1 mutation and CDKN2A/p16 delection
Yolk sac tumors	Multiple patterns: reticular/microcystic pattern most commonly and endodermal sinus pattern (Schiller-Duval bodies) most specific, cells with variable atyia	CK, SALL4, AFP, Glypican-3+; EMA, ER, PR, CD117, OCT4−	NS	NS
Signet-ring stromal tumors	Signet ring cells with rare mitotic count present in fibroma stromal background	CK focal+; CR, CyclinD1, β-catenin(nuclear), CD10, SF1, SMA+; EMA, inhibin-α−	PAS −; Mucin −	NS
Microcystic stromal tumors	A classic triad of microcysts, solid cellular zones, and fibrous stroma, cells with low mitotic activity	CK focal+; WT-1, CyclinD1, β-catenin (nuclear and cytoplasmic), CD10, SF1, FOXL-2 +; EMA, CR, inhibin-α, ER, PR −; AR+/−	NS	NS
Wolffian tumors	Four distinct patterns: diffuse or solid, tubular, retiform and multicystic, cells with low mitotic count	CK, Vim, CD10, AR +; EMA, ER, PR −/only focal +; GATA-3 −/ weak multifocal +; CK7, CR, WT-1, inhibin-α, FOXL-2 focal +; SF-1 −	PAS +	NS
Lymphangioma	Consist of cavernous or cystic dilated lymphatics	CK −; Vim, CD34, CD31, FVIII, D2-40 +	NS	NS

+, positive; −, negative; NS, not special

The typical histomorphologic feature of the 2 cases was the presence of signet-ring cells, which was necessary for the differential diagnosis of Krukenburg tumors. Krukenburg tumor is a malignant tumor formed by the metastasis of signet ring cell carcinoma of the stomach to the ovary. Histologically, it is characterized by signet-ring cells, often accompanied by the growth pattern of surface implantation, and it is easy to be confused with ATs. More obvious cellular atypia of Krukenburg tumors is the main diagnostic clue. In addition, the history of gastric cancer, elevated levels of tumor serum markers such as CEA, CA199, and IHC analysis may jointly contribute to differentiate it from ATs. Krukenburg tumors are positive not only for mucin staining but also for epithelial immunological indicators for example, EMA, CEA, MOC-31, and Ber-EP4. In our cases, the epithelial biomarkers were all negative, and Krukenburg tumors were excluded.

In the 2 cases, the mesothelial immunological indicator (Calretinin, CK5/6, WT-1, and D2-40) findings were positive, which was needed to make the differential diagnoses of well-differentiated papillary mesothelial tumor, a borderline tumor and mesothelioma, a malignant tumor. No papillary structure was present in the 2 cases, this excluded well-differentiated papillary mesothelial tumors, which have a typical feature of exophytic papillary hyperplasia. Mesotheliomas were ruled out, due to the lack of architectural complexity, cellular atypia, stromal invasion, and genetic alteration incompatibility.

Yolk sac tumors, which have structural similarity with ATs, were also one of the tumors that need to be identified. Yolk sac tumor is a highly heterogeneous malignant tumor and has a variety of histological structures. The present 2 cases lacked multiple patterns—especially Schiller-Duval bodies and remarkable cellular atypia. Moreover, SALL4 was negative. Yolk sac tumors could be excluded. Other differential diagnoses are described in Table 2.

## Conclusions

In our study, 2 cases of ovarian ATs were presented and extensive literature review, histopathological, IHC, histochemical stain, and FISH analyses were performed to improve our understanding of this rare tumor. In mechanism, ovarian ATs may not be related to BAP1 or CDKN2A/p16 mutations, which are characteristic of most mesotheliomas. In addition, the case 1 suggests that ATs may be associated with immune dysregulation. When encountering such similar lessions, we recommend that a series of immunohistochemical, histochemical and molecular biological techniques should be used for diagnosis and differential diagnosis. Both pathologists and clinicians need to improve the understanding of ovarian ATs. Pathologists should avoid misdiagnosis as ovarian malignancy especially in intraoperative frozen pathological diagnosis, and misleading clinicians to cause overresection. The limitation of the study is that it is a single-center case study with a small number of cases. More cases are needed to analyze its pathogenesis in the future.

## Data Availability

All available data generated and analyzed during this study are included in this published article.

## Abbreviations

ATs: Adenomatoid tumors
IHC: Immunohistochemistry
FISH: Fluorescence in situ hybridization
BAP1: BRCA1 associated protein 1
L1CAM: L1 cell adhesion molecule
AB-PAS: Alcian blue and periodate Schiff's reaction
CT: Computed tomography
MRI: Magnetic resonance imaging
CRP: C-reactive protein
TSH: Thyroid-stimulating hormone
TRAFs: Tumor necrosis factor receptor-associated factors
